# Prevalence, Phytochemical Investigation, and *In Vitro* Acaricidal Efficacy Evaluation of *Dodonaea angustifolia*, *Eucalyptus globulus*, *Millettia ferruginea*, and *Euphorbia abyssinica* against Sarcoptic Mange of Camel, Babile District, Ethiopia

**DOI:** 10.1155/2022/8639370

**Published:** 2022-08-11

**Authors:** Sisay Alemu, Yehualashet Bayu, Pawlos Wasihun, Abdallahi Abdurahman

**Affiliations:** Haramaya University, College of Veterinary Medicine, Ethiopia, P.O. Box 138, Dire Dawa, Ethiopia

## Abstract

The present study was designed to determine the prevalence of sarcoptic mange from camels; evaluate the presence of secondary metabolites in the methanolic leaf extracts of *D. angustifolia M. ferruginea*, *E. abyssinica*, and *E. globulus* essential oil; and also evaluate their *in vitro* acaricidal efficacy against camel sarcoptic mange in the Babile District, Ethiopia. Each plant was subjected to qualitative phytochemical screening for the presence or absence of secondary metabolites. The *in vitro* application of each plant extracts was carried out against clinical mange mites with positive and negative controls. Accordingl*y*, a total of 384 camels were examined for the presence of *Sarcoptes scabiei* var. *cameli.* The study revealed that an overall prevalence of 67(15.9%) animals was infested by mange mites. The study showed that a higher prevalence was observed in female camel than in male camel with no statistically significant difference (*P* > 0.05). However, statistically higher prevalence (*P* < 0.05) was recorded in young camel (28.2%) than adult (10.95%). The study also revealed that statistically higher prevalence (*P* < 0.05) was observed in poor body conditioned camel (26.3%) than medium (13.5%) and good body condition camels (5.4%). For the *in vitro* evaluation of the efficacy of plant extracts, essential oils and crude extracts of four medicinal plant extracts were diluted at different concentrations from 200 mg/mL to 6.25 mg/mL and added to Petri dishes containing the adult stage of *Sarcoptes scabiei*. The efficacy of each plant was determined by comparing the mortality of mites treated with plant extracts with those of mites in nontreated (2% Tween 80) and treated (diazinon) control plates. After 3 h of contact, all concentrations of *E. globulus*, *D. angustifolia*, *M. ferruginea*, and *E. abyssinica* showed good *in vitro* acaricidal efficacy compared to controls nontreated control (*P* < 0.05). After 3 h of exposure to the methanolic extract of *D. angustifolia* and the essential oil of *E. globulus*, comparable acaricidal efficacy in causing mites mortality at concentrations of 200 mg/mL has been recorded when compared to reference drugs. The crude extract of *E. abyssinica* showed a lower acaricidal efficacy compared to reference drugs. The present plant extracts studied showed comparable acaricidal efficacy to reference drugs at concentrations of 200 mg/mL. Therefore, further epidemiological and in vivo acaricidal studies as well as toxicity of the plants should be conducted in area to use these plants as alternative treatment option to substitute the use of synthetic drugs for which most of mites developed resistance and control impacts *Sarcoptes scabiei* on camel.

## 1. Introduction

Camels are adaptable animal species in ensuring food security and worthwhile the livelihood priorities of the pastoral community in the arid and semiarid areas of Ethiopia [1, 2]. Diseases, as well as other issues like poor diet and poor husbandry techniques, pose a threat to the region's ability to produce camels [3, 4]. Among the disease of camel, mange mites are common in Ethiopia [5] that causes significant economic loss of camel productivity [6]. Camel mange is one of the most common and economically significant zoonotic and epizootic diseases. It is an infectious ectoparasite that is caused by *Sarcoptes scabiei* var. *cameli.* It spreads among animals through direct physical contact with an infested animal and indirectly through fomites [4]. Clinically, it is manifested by hair loss, severe itch, and crusty or scaly skin lesions [7].

Treatment of mange with various acaricides like diazinon, fenvalerate, deltamethrin, and avermectin has been attempted with different grades of success. One of the main problems associated with the widespread use of synthetic acaricides is the quick emergence of resistance to them [8, 9], high cost and environmental contamination [10], and health hazards to humans during treatment of animal [11]. Concern about toxicity [10] of many acaricides restricts their use and lowers the level of safety of existing acaricide products. These problems have led to research efforts to discover new effective compounds.

The discovery of novel active plants derived from natural compounds may increase the number of chemotherapeutic agents that are readily available, thereby reducing the frequency of the emergence of resistance and bringing about a greater acceptance of alternative medicines, particularly in terms of environmental safety [12]. The use of botanical acaricides is crucial against highly pathogenic and economically significant ectoparasites like mange. The different indigenous plants such as *Cedrus deodara*, *Pongamia glabra*, *Diospyros scabra*, *Dobera glabra*, *Euphorbia abyssinica*, and *Sterculia alexandri* have been tried against mange mites from sheep [13]. In many parts of the world, mange mites in animals are treated using various components of several plants. However, there is limited information, and no studies are available on the efficacy of medicinal plants against economically important mange mites of camel in Ethiopia. Therefore, the present study was designed to determine the prevalence of mange mites and associated risk factors from camels in Babile District, Oromia regional state, Ethiopia, as well as to determine the secondary metabolites present in selected plant extract and to evaluate their *in vitro* acaricidal efficacy against sarcoptic mange from camels.

## 2. Materials and Methods

### 2.1. Study Area

**Figure 1:**
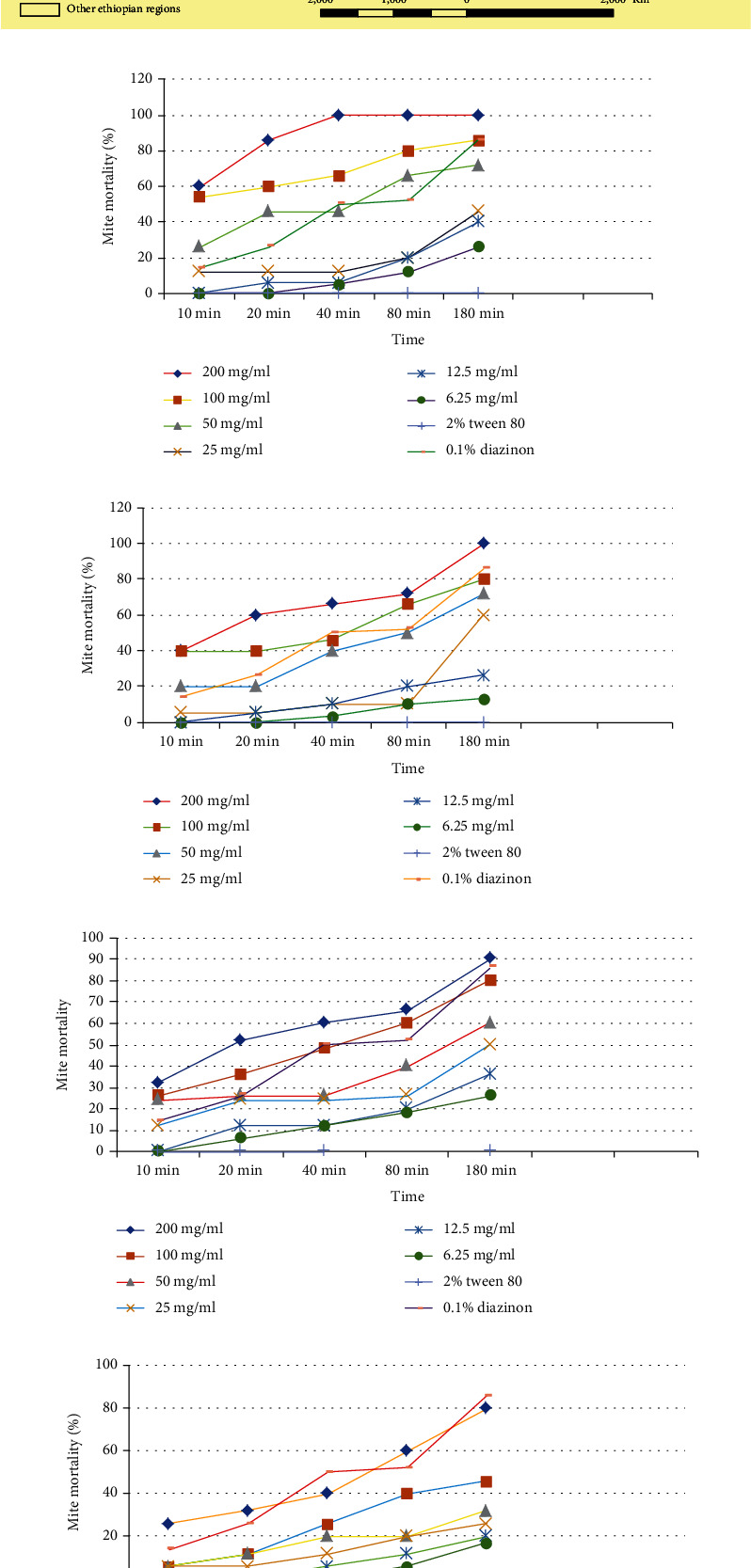
(a) Map representing the study area. (b) Mortalities of mites treated with the *Dodonaea angustifolia* crude extract. (c) Mortalities of mites treated with the *Eucalyptus globulus* essential oil extract. (d) Mortalities of mites treated with the *Millettia ferruginea* crude extract. (e) Mortalities of mites treated with the *Euphorbia abyssinica c*rude extract.

The study was conducted from September 2019 to November 2020 in three kebeles of Babile District in Oromia regional state, Ethiopia. The districts were selected purposely based on the camel population and access to transportation, Babile District is found in the eastern Hararghe zone of Oromia regional state, Ethiopia. It is located 31 km away from Harar town and about 557 km east from Addis Ababa, the capital city of Ethiopia. It lies between 8°, 9′9°, 23′ N latitude and 42°, 15′-42°, 53′ E longitude (Figure 1(a)) and is characterized by semiarid and arid climate with average annual rainfall of 410-800 mm and the annual temperature ranges from 24 to 28°C. It shares its border with Gursum from the north, Fedis from the west, and Harar National Regional State from the north-west and Somalia National Regional State in the East, South, and South West. In Babile District, there is an estimated total camel population of 12,000 and 300 camel owners in 21 rural kebeles [14].

### 2.2. Study Animals

Camels managed under an extensive management system were used for the present study. Mange mites, collected from camels in the study sites, were brought to Haramaya University Veterinary Laboratory for the experimental study.

### 2.3. Study Design

#### 2.3.1. Cross-Sectional Survey

Camels from three districts were randomly selected. The sample size for this study was determined using Thrusfield [15], accepted error 5%, and confidence level of 95%. Based on this, the sample size was calculated using the following formula.

(1) $$\begin{array}{c} \frac{n=1.962\times \text{Pexp }\left(1-\text{Pexp}\right)}{d2}, \end{array}$$

where *n* is required sample size, *P* is expected prevalence, and *d* is the level of precision (5%), 1.96 to indicate 95% confidence level. Therefore, based on the above formula, a total of 384 camels were examined to determine the prevalence, risk factors, and species of mange mites in the study area.

#### 2.3.2. Clinical Examinations

A total of 384 camels were randomly selected and clinically examined for the presence of skin lesions. Before the clinical examination, the animal age, sex, body condition score, and level of infestation was recorded. The degree of mange mite infestation was judged as described by Genchi et al. [16].

### 2.4. Parasite Collection, Transportation, and Identification

Mange mites were collected from a camel and exhibited clinical signs of the mange infestation such as hair loss, severe itch, and crusty or scaly skin lesions. The wool was clipped out with scissors and then drops of glycerin were added on the edge of skin lesions to moisten the area. *Sarcoptes scabiei* and Demodex species, two types of follicular and burrowing mites, can be diagnosed by deep skin scraping (deep epidermal examination), which is done until capillary ooze appears. In line with [17], a scraping was placed in a Petri dishes transport to HU-CVM parasitology laboratory. The sample were treated with 10% potassium hydroxide solution and examined under a light microscope. Identification of the species of mange mites were performed according to the key morphological characteristics described by [18]. The collected mites were placed in Petri dishes, and all the Petri dishes were incubated at 35°C for 30 min, according to [19]; after being identified under a stereomicroscope, adult mites with eight legs were used in the studies.

### 2.5. Plant Collection, Extraction, and Phytochemical Screening

The leaves of selected plants of *D. angustifolia*, *E. abyssinica*, and *E. globulus* were collected from their natural habitat, identified, and verified at the Haramaya University College of Agriculture Department of Plant Science.voucher No NOVLFEN/09/20, NOVLFEN/74/06, NOVLFEN/10/20, and NOVLFEN/11/20, respectively. *D. angustifolia*, *M. ferruginea*, and *E. abyssinica* leaves that had been air dried were powdered, and 400–500g of that was thoroughly extracted with methanolic solvent by percolation at room temperature. To get the crude extracts, the menstrual fluid was filtered and concentrated in a rotary evaporator. Until they were utilized in efficacy research, the concentrated extracts were stored in a firmly closed bottle in the refrigerator. The essential oil was extracted by subjecting the air-dried leaves of *Eucalyptus globulus* to hydro distillation using a Clevenger-type apparatus. In a 5-liter round bottom flask, 100g of powdered plant material was combined with distilled water. The hydro distillation process took three hours, and the obtained oil was dried with sodium sulfate before being refrigerated and stored at 4°C [20]. The extraction rate (%) will be calculated as follows [21].

(2) $$\begin{array}{c} \text{Extraction rate }\left(\%\right)=\frac{\text{Weight of extracts}\left(\text{g}\right)}{\begin{array}{c} \text{ Weight of the plant material}\left(\text{g}\right)\\ \text{before extraction} \end{array}}\times 100 \end{array}$$

#### 2.5.1. Phytochemical Screening of Solvent Extracts

The secondary metabolites of the extract, such as alkaloids, steroidal compounds, phenolic compounds, flavonoids, saponins, tannins, phlobatannins, triterpenes, and glycosides, were identified in a tiny amount of the extract/fraction [21].

Phytochemical screening was carried out to assess the qualitative chemical composition of crude extracts using commonly employed precipitation and coloration reaction to identify the major natural chemical groups and secondary metabolites present in the plants. Combinations of several methods were used to identify the phytochemicals of the medicinal plants. Standard screening tests were conducted using a conventional protocol and reagents on the methanolic extracts of herbs to identify the constituents as described by Sofowora [17]. The screening was conducted to detect for the presence of bioactive chemicals such as saponins, tannins, flavonoids, steroids, phenolic compounds, alkaloids, glycosides, and triterpenes that are thought to have acaricidal properties.

Test for saponins (frothing test): 300 mg extract was boiled with 5 mL water for two minutes. The mixture was cooled and mixed vigorously and left for three minutes. The formation of frothing indicated the presence of saponins.

Saponins test (frothing test): 5 mL of water and 300 mg of extract were heated for 2 minutes. After being thoroughly mixed and cooled, the mixture was left for three minutes. The formation of frothing indicated the presence of saponins.

Tannins (ferric chloride test): 0.5 g of the extract was boiled in 10 ml of water in a test tube and then filtered. A few drops of 0.1% ferric chloride (or dilute ferric chloride solution) were added brownish green or a blue-black coloration indicating the presence of tannins.

Test for flavonoids (Shinoda test): a steam bath was used to reheat 1.5 mL of 50% methanol after dissolving 0.5 g of the extract in it. Five drops of strong hydrochloric acid and five of metallic magnesium were added. The presence of flavonoids is indicated by a red or orange tint.

The Liebermann-Burchard test for steroids: 10 mL of HCl and an equal volume of concentration were used to dissolve 1 milligram of the extracts. The sulfuric acid layer should be yellow with green fluorescence as the upper layer gets red. This suggests that steroids are present.

Test for triterpenes (Liebermann-Burchard test): 5 mL of HCl and 300 mg of extract were combined and then heated for 30 minutes. Concentrated sulfuric acid was added in a few drops and then well combined. Triterpenes are present when the red color first appears.

In order to test for phenolic compounds, 2 mL of the aqueous macerate of the plant material was filtered, and 3 drops of a freshly prepared solution of 1 ml of 1 percent ferric chloride and 1 ml of potassium ferrocyanide were added. The development of a bluish-green color was taken as evidence that phenolic compounds were present.

Test for glycoside (FeCl_3_ test): 0.5 g of the extract/fraction and 5 mL of concentrated H_2_SO_4_ were added and boiled for 15 min. This was then cooled and neutralized with 20% KOH. A green to black precipitate indicated phenolic aglycone as a result of hydrolysis of glycoside.

Test for alkaloids (Dragendoffís test): a 100 mg of plant extract was dissolved in dilute hydrochloric acid. Solution was clarified by filtration. The film was tested with Dragendroff's and Mayer's reagents. Occurrence of orange-red precipitate indicates the presence of alkaloids.

Phlobatannins were detected by heating 2 mL of the aqueous extract in 1 percent HCl. The formation of a crimson precipitate was considered proof that phlobatannins were present.

### 2.6. *In Vitro* Acaricidal Efficacy Test

#### 2.6.1. Adult Immersion Test

Mites were collected from naturally infested animals. The scabs and scrapings from the mange-like lesion were incubated at 35°C for 30 min [22] and then observed under a stereomicroscope to isolate adult mites of both sexes. Then, mites were placed in a Petri dish (approximately five mites/dish) in three replications. The experiment involved mites with eight legs in their adult stages. In this study, from stock solution of each plants of methanolic crude extracts, a working solution in a total of 6 (six) dilutions were prepared using appropriate solvents. The solvent (2% Tween 80) were used to dilute the methanolic plant extracts, and Tween 80 and 1% diazinon were used as negative and positive controls, respectively.

The plant extracts were diluted to different concentrations using appropriate solvents as described by Fichi et al. [23]. A 250 *μ*L of each solution were directly added to Petri dishes. Three replicates were prepared for each concentration of the extracts. As nontreated control, three Petri dishes containing only 250 *μ*L of the solvents and three replicates of Petri dishes were treated with 2%Tween 80 while three Petri dishes containing 250 *μ*L of 0.1% diazinon 60% EC (diluted with water at 1 : 1000 by volume as described by the manufacturer). All Petri dishes were kept at room temperature for 10 minutes, 20 minutes, 40 minutes, 80 minutes, and three hours after the treatments were inoculated, and each mite in the Petri dishes was carefully examined for death under a stereoscopic microscope [24]. Following the administration of the treatments, all plates containing various concentrations of the plant extract as well as treated and untreated control plates were kept at room temperature and observed under a stereoscopic microscope at 10 minutes, 20 minutes, 40 minutes, 80 minutes, and three hours [24]. Regular needle stimulation to test the vitality of mites resulted in the mites being reported as dead if there was no reaction [25]. The total numbers of mites present were counted, and the % efficacy of each treatment was calculated as given by Khan et al. [26].

(3) $$\begin{array}{c} \frac{\%\text{efficacy}=\text{No}.\text{of mites before treatment}-\text{No}.\text{of mites after treatment}\times 100}{\text{No}.\text{of mites before treatment}}. \end{array}$$

### 2.7. Data Analysis

Collected raw data was stored in a Microsoft Excel database system used for data management. Statistical software package called SPSS windows version 20.0 was used for data analysis. Descriptive statistics, percentages, and 95% confidence intervals were used to summarize the proportion of infected and noninfected animals. The effects of different host risk factors were considered and computed. All statistical significant levels was set at *P* < 0.05, and analysis of variance (one-way ANOVA-Tukey test) was used to compare the means of different treatments (concentrations) of the extracts and controls in different time used for in vitro acaricidal efficacy studies of medicinal plants.

## 3. Results

### 3.1. Prevalence of Mange Mites

Of the total 384 animals examined for the infestation of mange mites, 67 (15.9%) camels were infested by mange mites (Table 1(a)). Among the risk factors assessed, comparative higher prevalence was observed in female camels 21 (16.1%) than in male camels 40 (15.8%), but there was no statistical significant difference (*P* > 0.05). The study revealed that there was statistical significant difference (*P* < 0.05) between young animals 31 (28.2%) and adult animals 30 (10.95%) (Table 1(a)). The present study also showed that body condition scores of camels had significant effect on the occurrence of mange mites infestation (*P* < 0.05) and higher rates of infestation was showed in poor body conditioned camel 34 (26.3%) compared to medium and good body condition camels 22 (13.5%) and 5 (5.4%), respectively (Table 1(a)).

### 3.2. Plant Extract Yield and Physicochemical Properties

The percentage of extracted yield and means of dilution have been depicted in Table 1(b). All methanolic leaves crude extract of *D. angustifolia*, *M. ferruginea*, *E. globulus*, and *E. abyssinica* were diluted in 2% Tween 80. Therefore, phytochemical analysis of selected different plant extracts was screened for the presence or absence of secondary metabolites such as alkaloids, saponins, phenol, phlobatannin, steroids, flavonoids, glycosides, tannins, and triterpenes (Table 1(c)). The phytochemical screening showed the presence of phenol and flavonoids in all plant extracts.

### 3.3. *In Vitro* Acaricidal Efficacy Evaluation of Plant Extracts

The acaricidal activity of four distinct medicinal plant extracts against mites was examined. Mortalities of mites treated with the different concentration of the crude extract of *D. angustifolia* are shown in Figure 1(b). The maximum acaricidal efficacy was observed at a concentration of 200 mg/mL of the extract, which resulted in 100% mortality after 40 minutes of exposure. However, after 180 minutes of exposure, the 100 mg/mL concentration indicated an 86.6% mortality rate. The lowest concentration, used at 6.25 mg/mL of crude extracts, caused 26% mortality of mites compared to others at 180 minutes. The 200 mg/mL concentrations of the crude extract of *D. angustifolia* after 3 hours of exposure showed comparable acaricidal efficacy (*P* > 0.05) to the reference drug (0.1% diazinon) and untreated controls (2.0% Tween 80). In vitro acaricidal activity of *D. angustifolia* essential oil was good at all doses compared to untreated controls.

Mortalities for the mite treated with the different concentration of the essential oil extract of *E. globulus* are shown in Figure 1(c). *E. globulus* essential oil extract was found a comparable effect against *Sarcoptes scabies* mites at 200 mg/mL concentration with positive control. But after 3 h of contact time, the plant extract showed no acaricidal efficacy (*P* > 0.05) except at 200mg/mL concentration compared to the reference drugs (0.1% diazinon). There was a statistically significant difference (*P* < 0.05) among the different concentration of the essential oil extracts of *E. globulus* in causing mortality of mites. When compared to all the concentrations of the essential oil extract of *E. globulus*, it shows that there was a statistically significance difference (*P* < 0.05) in causing mortality. In vitro acaricidal efficacy of E. globulus essential oil was good at all doses compared to untreated controls.

Mortality rates for mites treated with various concentrations of *M. ferruginea* crude extract are shown in Figure 1(d). When compared to the positive control, the extract showed significant (*P* < 0.05) effects against *Sarcoptes scabiei* at all contact times of 200 mg/mL concentration. The extract had the highest acaricidal efficacy with 90% mortality after 3 h of exposure. After 3 hours of exposure, there was no significant effect (*P* > 0.05) between all concentrations compared to the positive control. When compared to the nontreated control, all concentrations of the crude extract showed a statistically significant (*P* < 0.05) difference in the mortality of mites.

Mortalities for the mite treated with the different concentration of crude extracts of *E. abyssinica* are shown in Figure 1(e). Crude *E. abyssinica* extracts at concentrations of 200 mg/mL exhibited higher acaricidal activity (80%) after 3 h of exposure and was statistically significant (*P* < 0.05) higher mortality compared to other concentrations. After 3 h of exposure, the 200 mg/mL concentration of the crude extract *E. abyssinica* does not show significant acaricidal efficacy (*P* > 0.05) with 0.1% diazinon (the reference drug). All extract concentrations showed statistically significant difference (*P* < 0.05) to cause mortality of mites compared to nontreated control (2% Tween 80).

In vitro comparative acaricidal activity of different crude extracts of essential oil of *D. angustifolia*, *M. ferruginea*, *E. abyssinica*, and *E. globulus* has been conducted. After 10 min postexposure with different concentrations, *D. angustifolia* showed statistically significant (*P* < 0.05) higher mortality of 3.00 ± 0.57 than *M. ferruginea* (1.66 ± 0.57^b^) and *E. abyssinica* (1.33 ± 0.33^b^) (Table 1(d)). Similar to *D. angustifolia* (4.33 ± 0.33^a^), *E. globulus* (3.00 ± 0.00^ab^), and *M*. *ferruginea* (3.00 ± 0.00^ab^) showed a good in vitro mite killing effect at 200 mg/mL concentration after a 20-min exposure time comparable to the positive control (Table 1(e)). However, the mortality effect after 40 min of exposure to 200 mg/mL concentrations of crude extract of *D. angustifolia* showed (100%) significant (*P* < 0.05) acaricidal activity 5.00 ± 0.00^a^ comparing to *M. ferruginea* (3.00 ± 0.00^b^), *E. globulus* (3.33 ± 0.33^bc^), and *E. abyssinica* (2.00 ± 0.00^c^) (Table 1(f)). The results revealed that the crude extract of the plant *D. angustifolia* showed mortality of 4.00 ± 0.57^a^ that was significantly (*P* < 0.05) higher than the essential oil of *E. globulus* (3.33 ± 0.33^a^), *M. ferruginea* (2.66 ± .0.33^b^), and *E. abyssinica* (1.33 ± .0.57^c^) after 80 minutes of exposure at concentration of 100 mg/mL (Table 1(g)). The lower concentrations 25 mg/mL-6.25 mg/mL of *D angustifolia* (2.00 ± .0.57^a^), *M. ferruginea* (1.66 ± 0.88^a^), *E. abyssinica* (2.33 ± 0.33^a^), and *E. globulus* (1.66 ± 0.33^a^) showed significantly lower mortality compared to reference drug (0.1% diazinon) after 180-minute exposures (Table 1(h)) and significantly lower concentrations of 25 mg/mL-0.65 mg/mL of *D angustifolia* (2.00 ± .0.57^a^), *M. ferruginea*, (1.66 ± 0.88^a^) *E. abyssinica* (2.33 ± 0.33^a^), and *E. globulus* (1.66 ± 0.33^a^) as compared to the reference drugs (0.1% diazinon) (Table 1(h)).

## 4. Discussion

The results of the present study revealed that the prevalence of mange mite infestation in the examined camels was 15.9%. The current finding is supported by previous reports of 16.7% prevalence from Raya and Azebo Districts in Ethiopia [27]. On the other hand, this results are lower than a previous study report by Zeleke and Bekele [28] and Atmani-Merabet et al. [29] with prevalence of 35.4% and 55.2% at Addis Ababa and Sudan, respectively. These variations might be due to variations in the research area's biological settings and other husbandry practices. Animal age and body condition scores were shown to be statistically significant factors affecting camel mange infestation among the associated risk factors studied in this study. Furthermore, younger camels are more susceptible to mite infestation than older ones. Compared to camels in good body condition, it was discovered that low body condition made them more susceptible to mite infection. Additionally, the current finding was incongruent with a prior report by Zeleke and Bekele [28], which showed that younger camels with poor body conditions were more likely to have higher occurrences of mite infestations than older camels with good body conditions.

Phytochemical screening of the different selected plants extracts was conducted by using chemical tests to know the active ingredients present in crude and oil extract of various plants. The result showed that the leaf of *D. angustifolia* was found positive for alkaloids, saponins, phlobotanin, steroids flavonoids, glycosides, and phenolic compound, triterpens but negative for tannins. This finding was in agreement with the report by Lawal et al. [30] that shows the presence of alkaloids, saponins, phlobatannin, steroids, flavonoids, and phenolic compound, on methanol leaf extract of *D. angustifolia*. However, another study by Deressa et al. [31] showed that leaf of *D. angustifolia* contains several secondary metabolites such as quinines, saponins, flavonoids, alkaloids, terpenoids, diterpenoids, phenols, and essential oils. Similarly, methanolic leaf crude extract of *D. angustifolia* was positive for alkaloids, quinines, saponins, terpenoids, diterpenoids, and phenols reported by Mengistie et al. [32]. Meanwhile, another previous study reported by Anode et al. [33] showed that the leaf extract of *D. angustifolia* were positive for alkaloids and flavonoids. Furthermore, the present results were in consistent with previous reports of Janzen [34] in which the leaf extract of *D. angustifolia* mainly has flavonoids, alkaloids, flavonoids, glycosides, terpenoids, anthraquinones, essential oils, and saponins.

The chemical analysis of the methanolic leaf oil extract of *E. globulus* was found positive for saponins, phlobatannin, glycosides, tannins, phenolic compounds, and triterpenes and negative for alkaloids. This finding was in line with the report of Syukri et al. [35], in which methanolic leaf extract of *E. globulus* showed high content of saponins, steroids, flavonoids, glycosides, tannins, and phenolic compound. Other study conducted by Parekh and Chanda [36] and Kaur et al. [37] showed that aqueous leaf extract of *E. globulus* was found positive for tannins, saponins, and phenolic compound. Despite this consistency, presence of tannins, alkaloids, saponis, glycosides, steroids, terpenoides and phenolic compounds [37]. Moreover, phytochemical screening of leaf extracts of *E*. *abyssinica* was positive for saponins, phenol, flavonoids, and tannins but negative for alkaloids, phlobatannin, steroids, glycosides, and triterpenes in the present finding. This finding is comparable with the report of Koul et al. [38], in which methanolic leaf extract of *E. abyssinica* was positive for saponins, tannins, and phenolic compound. Furthermore, our present results of the chemical analysis of leaf extract of *M. ferruginea* revealed positive for alkaloids, phlobatannin, steroids, flavonoids, glycosides, tannins, triterpenes, and phenolic compound but negative for saponins. Therefore, this study was in line with the report of Kumar et al. [39] that *M. ferruginea* crude extract contains alkaloids, phlobatannin, steroids, glycosides, phenol, and triterpenes. Similarly, Kumar et al. [6] reported that *M. ferruginea* seed oil has a flavonoid.

The current study on *in vitro* acaricidal efficacy activity of different extracts revealed that crude extract of *D. angustifolia* showed 100% mortality. The pronounced acaricidal activity of complete immobility of the *Sarcoptes scabiei* was recorded at a concentration of 200 mg/mL after 40-min exposure. This finding is in line with the report by Amelo et al. [40] and Amelo et al. [40] that aqueous extracts of *D. angustifolia* exhibited acaricidal efficacy higher than 90% against *T. urticate*. Methanolic root extract of *D. angustifolia* showed that 84.52% suppression of hemoparasite [41] and aqueous leaf extract of *D. angustifolia* revealed 35.79% against plasmodium [42]. The presence of flavanol glycosides in *D. angustifolia* contributed to the anthelminthic property [43]. The presence of phenol in this plant makes antioxidative activity that can inhibit haem polymerization in which the unpolymerized haem is very toxic to the parasite.

The essential oil extracts of *E. globulus* showed 100% mortality at a concentration of 200 mg/mL at 3-h exposure. Similarly, Dehghani-Saman et al. [44] reported that *E. globulus* cause 100% mortality by 20% concentration against *B. microplus* [45]. The essential oil extract of *E. globulus* exhibited 90% acaricidal activity against *Dermanyssus gallinae* and 76% mortality against *Rh Bursa* [46]. The extract of *E. globulus* showed significant acaricidal potential against *V. destructor* [l]. The extract of *E. globules* also revealed lousecidal effect on *P. humanuscapitis* [47], glycosides compound compounds present in *E. globulus* oil enhancing the cidal activity [48]. In addition, saponin, phenolic, and flavonoid are also present in crude extracts of this plant. These bioactive compounds have the ability to disrupt cell walls of the parasite and cause mortality [49].

The results obtained in the present study indicated the *M. ferruginea* leaf extract (90%) mortality of *Sarcoptes scabiei.* This finding supported by Choudhurt et al. [50], who reported that 100 mg/mL concentration of *M. ferruginea* seed oil caused 100% mortality of Boophilus decoloratus after 5 h contact [6]. Likewise, 20-80% concentrations of *M. ferruginea* seed oil showed 100% mortality against *Amblyomma variegatum* larvae. In the current study, *E. abyssinica* crude extract of 200 mg/mL concentration showed 80% mortality at 3-h postexposure. This could be due to the fact that cidal activity was attributed to the presence of secondary metabolites such as steroid and triterpene [51].

The results of the present study revealed that *D. angustifolia* crude extract showed that 100% mortality was significantly (*P* < 0.05) higher than to *E. globulus* essential oil (72%), *M. ferruginea* (66%), and *E. abyssinica* (60%) after 80 minutes of exposure at 100 mg/mL concentrations. However, after 3 h of exposure, the crude extract *D. angustifolia* and essential oil of *E. globulus* showed 100% mortality at a concentration of 200 mg/mL. However, there were no statistically significant difference (*P* > 0.05) in the acaricidal activity observed. Furthermore, 200 mg/mL of crude leaf extract of *D. angustifolia* exhibited statistically significant high acaricidal activity (*P* < 0.05) after 10-180 minutes of exposure compared to *E. abyssinica.* The acaricidal effect of the plant extract depends on the dose, time, and type of plant species. Furthermore, the highest mortality effect against *Sarcoptes scabiei* was observed by the crude extract of *D. angustifolia* and *E. globulus* essential oil. Relatively lower (26%) acaricidal activity (*P* < 0.05) was observed in 35 mg/mL concentration of E. abyssinica extract when compared to reference drug (0.1% diazinon). Even the higher concentrations (100 mg/mL and 50 mg/mL) observed lower acaricidal efficacy (46% and 32%), respectively, which is much lower to say effective. The higher or lower acaricidal activity could attribute to the presence or absence of secondary metabolites such as alkaloid, glycosides, and phenol when present and gave antiacaricidal activity [52]. A study indicates that the presence of alkaloid glycosides and phenol is an important chemical to initiate the mechanism of action *in vitro* and *in vivo*, acaricidal activity causing mortality against acarine [6]. In general, along with the economic benefits, an additional advantage of using plant pesticides is that they have low environmental persistence, do not induce resistance readily in insects, and are relatively nontoxic to mammals. These results consolidate the belief that the use of herbal acaricides can provide a better way to combat a menace such as mange in domestic animals and can be used more safely and effectively [53].

## 5. Conclusions

The current study showed that mites are the most prevalent ectoparasites of camels in the study area. Animal age and body condition scores of the animals are among the main associated risk factors attributed to the prevalence and distribution of mange mites in the study area. The results of the phytochemical screening test compared to acaricidal activity showed that the more secondary metabolites plant extracts have the more effective against mange mite mortality. This indicated that secondary metabolites have a synergistic effect on mite mortality. All medicinal plants showed *in vitro* acaricidal efficacy comparable to reference drugs at high concentration. The present study indicated that the crude extract and essential oils of the extracts tested could represent a possible alternative for the treatment of sarcoptic mange in camel. In addition, epidemiological studies should be conducted in the area to determine economic impacts due to mange mites from camels and other species of animals. Thus, further studies should be carried out on the isolation and characterization of the responsible active components of the selected plant materials and on drug formulation of their safety and *in vivo* efficacy, as well as cost effectiveness of the products that exhibited strong acaricidal activity, with the aim of substituting conventional synthetic acaricides. The low efficacy of diazinon should be further investigated to elucidate the activity range of the compound and the suspected prevalence of resistance by not only sarcoptic mange mites but also other species of mange mites.

## Figures and Tables

**Table 1a:** (a) The overall Prevalence of mange mites based on different risk factors

Risk factors	Categories	No. examined animal	No. positive animal	Prevalence	*χ* ^2^	*P* value
Kebeles	Daketa	128	15	11.7	2.612^a^	0.271
	Anod	128	24	18.8		
	Mullu	128	22	17.2		
	Total	384	61	15.9		
Sex	Female	131	21	16.1	0.003^a^	0.532
	Male	253	40	15.8		
	Total	384	61	15.9		
Age	Adult	274	30	10.95	17.445^a^	0.001
	Young	110	31	28.2		
	Total	384	61	15.9		
Body condition	Poor	129	34	26.4	29.90^a^	0.001
	Medium	163	22	13.5		
	Good	92	5	5.4		
	Total	384	61	15.9		

**Table 1b:** (b) Percentage yield of crude/oil extracts of the selected medicinal plants leaf

Plants	Plant part extracted	Solvents for extraction	Weight of dry powder (g)	Weight of dry extract (g)	Yield %	Means of dilution
*D. angustifolia*	Leaf	Methanol	500	73	14.6	2% Tween 80
*M. ferruginea*	Leaf	Methanol	500	97	19.4	2% Tween 80
*E. globulus*	Leaf	Distilled water	500	38	7.6	2% Tween 80
*E. abyssinica*	Leaf	Methanol	400	69	17.25	2% Tween 80

**Table 1c:** (c) Qualitative determinations of active ingredients in crude/oil extracts of the selected plants

Secondary metabolites	*D. angustifolia*	*M. ferruginea*	*E. globulus*	*E. abyssinica*
Saponin	**+**	**—**	**+**	**+**
Tannin	**—**	**+**	**+**	**+**
Phenol	**+**	**+**	**+**	**+**
Steroids	**+**	**+**	**+**	**—**
Flavonoids	**+**	**+**	**+**	**+**
Phlobatannin	**+**	**+**	**+**	**—**
Glycosides	**+**	**+**	**+**	**—**
Triterpenes	**+**	**+**	**+**	**—**
Alkaloids	**+**	**+**	**—**	**—**

Note: +: present, -: absent (negative).

**Table 1d:** (d) Comparisons on acaricidal activity of different extracts on mange mites after 10-min exposure

Dose (mg/mL)	Mean number of mite dead (mean of mortality ± SE) post exposure			
	*D. angustifolia*	*M. ferruginea*	*E. globulus*	*E. abyssinica*
200	3.00 ± 0.57^a^	1.66 ± 0.57^b^	2.00 ± 0.00^ab^	1.33 ± 0.33^b^
100	2.66 ± 0.66^a^	1.33 ± 0.33^ab^	2.00 ± 0.00^ab^	0.33 ± 0.57^b^
50	1.33 ± 0.33^a^	0.66 ± 0.33^a^	1.00 ± 0.00^a^	0.33 ± 0.57^a^
25	0.66 ± 0.33^a^	0.00 ± 0.00^a^	0.33 ± 0.33^a^	0.33 ± 0.33^a^
12.5	0.00 ± 0.00^c^	0.00 ± 0.00^c^	0.00 ± 0.00^c^	0.00 ± 0.00^c^
6.25	0.00 ± 0.00^c^	0.00 ± 0.00^c^	0.00 ± 0.00^c^	0.00 ± 0.00^c^
2% Tween 80	0.00 ± 0.00^d^	0.00 ± 0.00^d^	0.00 ± 0.00^d^	0.00 ± 0.00^d^
1% diazinon	0.66 ± 0.33^d^	0.66 ± 0.33^d^	0.66 ± 0.33^d^	0.66 ± 0.33^d^

**Table 1e:** (e) Comparisons on acaricidal activity of different extracts on mange mites after 20-min exposure

Dose (mg/mL)	Mean number of mite dead (mean of mortality ± SE) postexposure			
	*D. angustifolia*	*M. ferruginea*	*E. globulus*	*E. abyssinica*
200	4.33 ± 0.33^a^	3.00 ± 0.00^ab^	3.00 ± 0.00^ab^	1.66 ± 0.66^b^
100	3.00 ± 0.57^a^	2.33 ± 0.33^ab^	1.66 ± 0.33^ab^	0.66 ± 0.33^b^
50	2.33 ± 0.33^a^	1.00 ± 0.57^a^	1.66 ± 0.33^a^	0.66 ± 0.66^a^
25	0.66 ± 0.33^a^	1.00 ± 0.57^a^	0.33 ± 0.33^a^	0.33 ± 0.33^a^
12.5	0.33 ± 0.33^a^	0.00 ± 0.00^a^	0.33 ± 0.33^a^	0.33 ± 0.53^a^
6.25	0.00 ± 0.00^c^	0.00 ± 0.00^c^	0.00 ± 0.00^c^	0.00 ± 0.00^c^
2% Tween 80	0.00 ± 0.00^c^	0.00 ± 0.00^c^	0.00 ± 0.00^c^	0.00 ± .0.00^c^
1% diazinon	1.33 ± 0.88^c^	1.33 ± 0.88^c^	1.33 ± 0.88^c^	1.33 ± 0.88^c^

**Table 1f:** (f) Comparisons on acaricidal activity of different extracts on mange mites after 40-min exposure

Dose (mg/mL)	Mean number of mite dead (mean of mortality ± SE) postexposure			
	*D. angustifolia*	*M. ferruginea*	*E. globulus*	*E. abyssinica*
200	5.00 ± 0.00^a^	3.00 ± 0.00^b^	3.33 ± 0.33^bc^	2.00 ± 0.00^c^
100	3.33 ± 0.33^a^	2.66 ± .0.33^b^	2.33 ± 0.33^b^	1.33 ± 0.33^c^
50	2.33 ± 0.33^a^	1.33 ± 0.88^b^	2.00 ± .0.57^c^	1.00 ± 0.57^bd^
25	0.33 ± 0.33^a^	0.33 ± 0.33^a^	0.66 ± 0.33^a^	0.66 ± 0.33^a^
12.5	1.00 ± 0.57^a^	0.33 ± 0.33^a^	0.66 ± 0.57^a^	0.33 ± 0.33^a^
6.25	0.66 ± 0.33^a^	0.33 ± 0.33^a^	0.33 ± 0.33^a^	0.00 ± 0.00^a^
2% Tween 80	0.00 ± 0.00^d^	0.00 ± .0.00^d^	0.00 ± 0.00^d^	0.00 ± 0.00^d^
1% diazinon	2.00 ± 0.57^d^	2.00 ± .0.57^d^	2.00 ± 0.57^d^	2.00 ± 0.57^d^

**Table 1g:** (g) Comparisons on acaricidal activity of different extracts on mange mites after 80-min exposure

Dose (mg/mL)	Mean number of mite dead (mean of mortality ± SE) postexposure			
	*D. angustifolia*	*M. ferruginea*	*E. globulus*	*E. abyssinica*
200	5.00 ± 0.00^a^	3.00 ± 0.57^b^	3.66 ± 0.33^b^	2.00 ± 0.57^c^
100	4.00 ± 0.57^a^	2.66 ± .0.33^b^	3.33 ± 0.33^b^	1.33 ± .0.57^c^
50	3.33 ± 0.66^a^	2.00 ± 0.88^b^	3.00 ± 0.57^ab^	1.00 ± 0.57^c^
25	1.00 ± .0.57^d^	0.66 ± 0.33^d^	0.66 ± 0.33^d^	0.66 ± 0.33^d^
12.5	0.66 ± 0.66^d^	0.66 ± 0.66^d^	0.33 ± 0.33^d^	0.33 ± 0.33^d^
6.25	0.66 ± 0.66^c^	0.33 ± 0.33^c^	0.33 ± 0.33^c^	0.00 ± 0.00^c^
2% Tween 80	0.00 ± 0.00^c^	0.00 ± .0.00^c^	0.00 ± 0.00^c^	0.00 ± .0.00^c^
1% diazinon	2.66 ± .0.33^c^	2.66 ± .0.33^c^	2.66 ± 0.33^c^	2.66 ± 0.33^c^

**Table 1h:** (h) Comparisons on acaricidal activity of different extracts on mange mites after 180-min exposure

Dose (mg/mL)	Mean number of mite dead (mean of mortality ± SE) postexposure			
	*D. angustifolia*	*M. ferruginea*	*E. globulus*	*E. abyssinica*
200	5.00 ± 0.00^a^	4.33 ± 0.33^a^	5.00 ± 0.00^a^	4.00 ± 0.00^a^
100	4.33 ± 0.33^b^	4.00 ± 0.00^b^	4.00 ± 0.00^b^	3.33 ± .0.33^b^
50	3.66 ± 0.33^c^	3.00 ± .0.00^c^	3.66 ± .0.33^c^	1.66 ± 0.88^c^
25	2.00 ± .0.57^a^	1.66 ± 0.88^a^	2.33 ± 0.33^a^	1.66 ± 0.33^a^
12.5	2.00 ± .33^d^	1.33 ± .88^d^	2.00 ± .0.57^d^	0.66 ± .0.66^d^
6.25	1.00 ± 0.57^c^	0.66 ± 0.33^c^	0.66 ± .0.33^c^	0.33 ± 0.33^c^
2% Tween 80	0.00 ± 0.00^c^	0.00 ± 0.00^c^	0.00 ± .0.00^c^	0.00 ± 0.00^c^
1% diazinon	4.33 ± 0.33^c^	4.33 ± 0.33^c^	4.33 ± 0.33^c^	4.33 ± .0.33^c^

## Data Availability

Data used to support the findings of this study are available from the corresponding author upon request.
